# Influence of systemic inflammatory indices on hospital stay and dialysis post-earthquake: A clinical study

**DOI:** 10.1371/journal.pone.0299737

**Published:** 2024-02-28

**Authors:** Burak Yasar, Pınar Ozbilgehan, Mert Sen, Arslan Guvendik

**Affiliations:** Department of Plastic, Reconstructive and Aesthetic Surgery, Ankara Bilkent City Hospital, Health Sciences University, Ankara, Türkiye; West China Hospital of Sichuan University, CHINA

## Abstract

Natural disasters pose significant challenges to medical response due to the surge of patients and emergent injuries. Amid such scenarios, where personnel for patient monitoring might be scarce, effective biomarkers are crucial for guiding treatment plans and predicting patient prognosis. We aimed to evaluate the correlation between systemic inflammatory indices and morbidity in earth-quake-induced crush injuries. Additionally, we assessed the potential of these indices as prognostic markers for adverse outcomes. We studied 140 patients with earthquake-related crush injuries (ECR) admitted between February and March 2023 and compared them to 200 healthy controls (CG) chosen using a simple random method. Using the complete blood count data upon admission, we computed and statistically compared indices including NLR (neutrophil lymphocyte ratio), PLR (platelet lymphocyte ratio), MLR (monocyte lymphocyte ratio), SII (systemic immune-inflammatory index), SIRI (systemic inflammatory response index), and PIV (pan-immune inflammation value). Regression analyses determined the prediction of hospitalization duration and dialysis necessity. PLR and MLR upon admission significantly predicted the length of hospital stay. MLR and SIRI were significant predictors for dialysis requirement, with Exp(B) values of 0.306 (p = 0.024) and 1.261 (p = 0.038), respectively. Systemic inflammatory indices can serve as valuable prognostic tools in disaster scenarios. Utilizing these indices can enhance patient management, effectively allocate resources, and potentially save lives in the aftermath of earthquakes.

## Introduction

On February 6, 2023, southeastern Turkey was struck by two major earthquakes, measuring 7.8 and 7.6 on the Richter scale, occurring just 9 hours apart [1]. These devastating events led to the loss of over 40,000 lives and left more than 100,000 injured [2]. The aftermath saw a surge in admissions to emergency departments and trauma-specific surgical units throughout the country. The impact also resonated with the local health infrastructure. A significant proportion of these injuries were high-energy crush injuries, which are typical following earthquakes [3]. Early detection and appropriate management of these injuries are crucial due to their high mortality and morbidity rates [3]. At times, medical professionals must make immediate decisions about fasciotomy or amputation [4]. In disaster scenarios, where the patient volume surpasses the available health personnel’s capacity for close monitoring, alternative biomarkers to clinical observation are essential to guide treatment plans and predict patient outcomes.

A Complete Blood Count (CBC) is among the primary laboratory tests administered to trauma patients. It offers insights into the patient’s hemoglobin level, erythrocyte count, platelet count, and various immune system cell subtypes. Presently, systemic inflammatory indices derived from CBC parameters are growing in importance as prognostic indicators for diseases. Indices like Neutrophil Lymphocyte Ratio (NLR), Platelet Lymphocyte Ratio (PLR), Systemic Immune-Inflammation Index (SII), Systemic Inflammatory Response Index (SIRI), Pan-Immune Inflammation Value (PIV), and Monocyte Lymphocyte Ratio (MLR) have been used to determine prognosis in conditions like cancer, cardiovascular diseases, and respiratory distress syndrome [5–7]. Yet, their potential in predicting morbidity from earthquake-induced crush injuries remains uncharted territory.

In this study, our objectives are twofold: to explore the association between systemic inflammatory indices and the morbidity arising from earthquake-induced crush injuries, and to discern if these indices can serve as prognostic markers for such injuries, especially in predicting unfavorable outcomes. Our findings could significantly impact disaster response strategies, enabling the early identification of high-risk patients and allowing for better resource allocation and treatment prioritization.

## Material and method

Our study is an observational, retrospective, cohort study. All participants provided informed consent, and all protocols were approved by the Ethics Committee of Ankara Bilkent City Hospital (Approval Number: E1-23-3539). We adhered strictly to the Declaration of Helsinki. Authors could not access to information that could identify individual participants during or after data collection. Data were accessed for research purposes between July 2023 and August 2023. Patients with earthquake and related injuries (EG) who were admitted to our clinic between February 2023 and March 2023 with the need for fasciotomy and body defects due to earthquake and related injuries and whose follow-up and treatment were performed by our clinic were included in our study.

A total of 340 patients, 140 (82 M, 68 F) with earthquake-related crush injuries and 200 (100 M, 100 F) controls were included in this study. Upon patient admission, a comprehensive set of data was recorded: age, gender, type and location of injury, duration under debris, time to hospital arrival, total injured sites, number and nature of amputations, dialysis sessions during hospitalization, quantities of erythrocyte suspensions, PLT transfusions, and TDP transfusions received. Additionally, overall hospital stay duration, and time taken to resume daily life were noted. From the CBC results at the time of presentation, counts for neutrophils (N), lymphocytes (L), platelets (P), and monocytes (M) were evaluated. For the control group (CG), preoperative NLR, PLR, MLR, SII, SIRI, and PIV values from 200 aesthetic surgery patients (comprising 100 females and 100 males) without known illnesses from our clinic served as the reference for a healthy population.

NLR using absolu peripheral neutrophil (N, x10^9^ /L) and lymphocyte (L; x10^9^/L) counts: NLR= N/L is calculated by the formula [8]. PLR is calculated using peripheral platelet (P; x10^9^/L) and lymphocyte counts: PLR=P/L [9]. MLR is calculated using peripheral monocyte and lymphocyte counts: MLR= M/L is calculated using the formula [10]. SII is calculated using peripheral platelet, neutrophil and lymphocyte counts: SII= (PxN) /L formula [11]. SIRI was calculated using peripheral neutrophil, monocyte and lymphocyte counts: SIRI= (N x M)/L. [12]. PIV is calculated using peripheral platelet, neutrophil, monocyte and lymphocyte counts: PIV= (P x N x M)/L [13]. All inflammatory markers are ratios or indices and therefore do not represent a unit. All inflammatory marker calculations were made using values at the time of initial presentation.

### Statistical analysis

Statistical evaluation of the data was performed using SPSS software, version 22.0 (IBM SPSS Statistics, IBM Corporation, Armonk, NY). Non-normally distributed data were evaluated by Kolmogorov-Smirnov test; p<0.05 was considered statistically significant. Data were presented as mean and standard deviation or median and range. Demographic percentages and mean measurements were compared between the two groups using the Mann-Whitney U test, chi-square test and Wilcoxon rank test. Pearson and Spearmen correlation tests were used where appropriate. Receiver operating characteristic (ROC) analysis was used to calculate optimal cut-off values, sensitivity, and specificity for systemic inflammatory markers to predict post-traumatic morbidity. For multivariate analysis, possible factors identified by univariate analyses were further entered into logistic regression analysis to identify independent predictors of morbidity. In all statistical analyses, p<0.05 was considered significant.

## Results

The study comprised 140 patients suffering from crush injuries due to an earthquake (82 males and 68 females), alongside 200 control subjects (equally divided with 100 males and 100 females). The mean age was 35 (min: 18, max: 53) in the control group (CG) and 36 (min: 3, max: 88) in the group with earthquake-related crush injury (EG): 36 (min: 3, max: 88).

These patients spent an average of 23 hours under debris before rescue, and it took an average of 68 hours to reach a hospital post-rescue. On average, patients had injuries across 1,8 different areas, with a total of 24 patients requiring amputations, amounting to 32 limbs. Dialysis was a common treatment, with an average of 1,6 sessions per patient and a total of 201 sessions recorded. Additionally, the average erythrocyte suspension replacements were notable at 6,1 per patient (850 in total), with TDP and platelet replacements averaging at 1,7 (355 total) and 0,09 (54 total) respectively. The average hospital stay was 52 days, and the study reported 6 fatalities among the patients. This comprehensive data underscores the severe impact of earthquake-related injuries on affected individuals and the extensive medical care required to address their conditions.

Demographic characteristics of EG are shown in **Table 1**.

**Table 1:** Demographic data of the group with earthquake-related crush injury.

Demographics	Earthquake-Related Crush Injury Group (n: 140)
**Age**	Average: 36 (min: 3, max: 88)
**Gender**	82 Male
	68 Female
**Time under debris (hours)**	Average 23 hours
	(min: 0, max: 97)
**Time to reach hospital (hours)**	Average 68 hours
	(min: 3, max: 280)
**Number areas of injured**	Average 1,8
	(min: 1, max: 6)
**Number of Amputations**	32 limbs
	Total: 24 patients
**Dialysis Sessions (average)**	Average: 1,6
	(min:0, max: 60)
	Total: 201
**Erythrocyte Suspension Replacements**	Average: 6,1
	(min: 0, max: 60)
	Total: 850
**TDP Replacements**	Average 1,7
	(min: 0, max: 38)
	Total: 355
**Platelet Replacement Count**	Average 0,09
	(min: 0, max: 3)
	Total: 54
**Length of Hospitalization (Days)**	Average:52 days
	(min: 1, max: 105)
**Number of Exitus**	6

NLR, PLR, MLR, SII, SIRI and PIV were significantly higher in EG. And this elevation was found to be statistically significant **(Table 2**).

**Table 2:** Results of systemic inflammatory indices according to groups.

Variebles	Control Group n: 200	Earthquake Related Crush Injury Group n: 140	p Value
NLR	2,48	9,5	**<0,001** *
PLR	136,9	262,9	**<0,001** *
MLR	0,37	0,69	**<0,001** *
SII	651,2	2898,1	**<0,001** *
SIRI	1,91	4,92	**<0,001** *
PIV	476,9	2744,3	**<0,001** *

*Statistically significant

Abbreviations: MLR, monocyte-to-lymphocyte ratio; NLR, neutrophil-to-lymphocyte ratio; PIV, pan-immune inflammation value; PLR, platelet-to-lymphocyte ratio; SII, systemic immune-inflammation index; SIRI, systemic inflammation response index.

**Fig 1:**
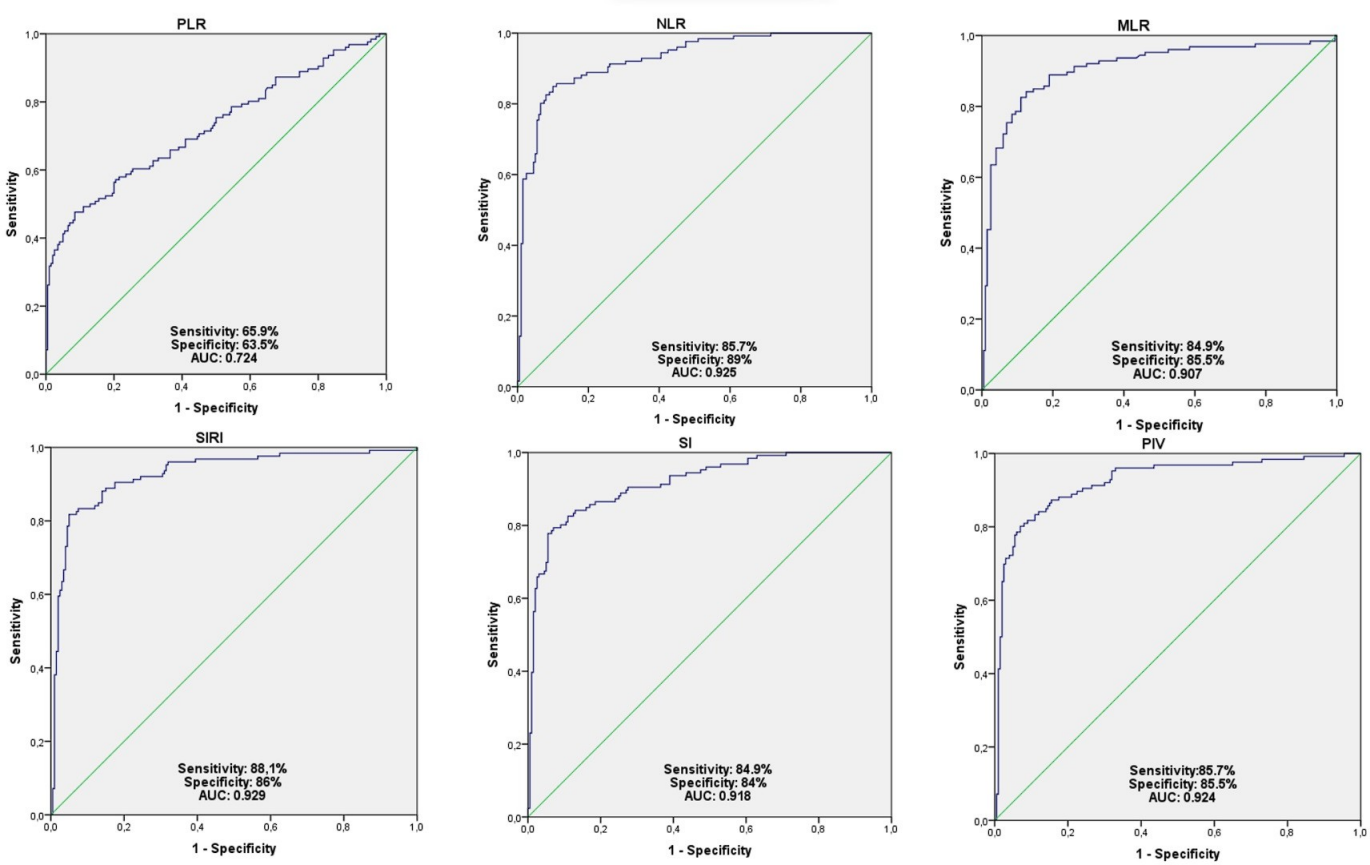
Receiver characteristic curve (ROC) to predict prognosis of earthquake related injuries.

Following ROC analyses, area under the curve (AUC) and 95% confidence interval values are shown in **Table 3**. The optimal cut off values for maximum sensitivity and specificity for NLR, PLR, MLR, SII, SIRI and PIV values are 3.47, 148.8, 3.47, 862.4, 1.52, 414.7, respectively (**Fig 1**).

**Table 3:** Sensitivity, specificity, and AUC of NLR, PLR, MLR, SII, SIRI, and PIV in earthquake related crush injury group.

Cutoff Value	Sensitivity	Specifity	AUG	95% CI	p	Lower Upper
NLR	3,47	85,7	89	0.925	0,896 0,955	**0.0001** *
PLR	148,8	65,9	63,5	0,724	0,664 0,783	**0.0001** *
MLR	0,28	84,9	85,5	0,907	0,870- 0,994	**0.0001** *
SI	862,4	84,9	84	0,918	0,887-0,950	**0.0001** *
SIRI	1,52	88,1	86	0,939	0,897-0,96	**0.0001** *
PIV	414,71	85,7	85,5	0,924	0,892-0,956	**0.0001** *

* Indicates statistically significant p-Values.

Abbreviations: AUG, Area under curve; CI, confidence interval MLR, monocyte-to-lymphocyte ratio; NLR, neutrophil-to- lymphocyte ratio; PIV, pan-immune-inflammation value; PLR, platelet-to-lymphocyte ratio; SII, systemic immune- inflammation index; SIRI, systemic inflammation response index. p-Value of receiver operating characteristic analysis that shows the significant sensitivity and specificity of these markers at reported cutoff values.

An analysis of variance (ANOVA) was performed to assess the significance of the linear regression model predicting duration of hospital stay based on several predictor variables. The regression model was found to be statistically significant (F(12, 113) = 3.149, p < 0.001), indicating that the model, as a whole, provides a significant improvement over an intercept-only model.

The individual predictors included in the model were fresh frozen plasma replacement, SI, number of amputations, number of injured areas, number of dialysis performed, PIV, PLR, Erythrocyte replacement, NLR, platelet replacement, MLR and SIRI. The results suggest that PLR and MLR on admission are statistically significant predictors of hospital stay. PLR had a significant positive effect on hospital stay (β = 0.058, t = 2.089, p = 0.039). MLR having a negative effect (β = -28.639, t = -2.109, p = 0.037). Number of amputations also appears to be a significant predictor with a positive effect. The other independent variables do not appear to have statistically significant effects on hospital stay (**Table 4).**

**Table 4:** Results of linear regression model performed to predict duration of stay based on several predictor variables.

Coefficients of Hospital Stay						
Model		Unstandardized Coefficients		Standardized Coefficients	t	p value
		B	Std. Error	Beta		
1	(Constant)	41,160	6,094		6,754	,000
	NLR	,095	1,044	,026	,091	,927
	PLR	,058	,028	,522	2,089	**,039** *
	MLR	-28,639	13,578	-,718	-2,109	**,037** *
	SI	-,004	,003	-,374	-1,155	,251
	SIRI	1,531	1,181	,490	1,296	,198
	PIV	,003	,002	,396	1,342	,182
	Number of injured areas	-1,023	2,674	-,035	-,383	,703
	Number of amputations	15,894	4,492	,321	3,538	**,001** *
	Number of dialysis performed	-1,112	,651	-,228	-1,709	,090
	Erythrocyte replacement	,688	,464	,269	1,482	,141
	Platelet replacement	-,736	1,788	-,084	-,412	,681
	Fesh frozen plasma replacement	-,351	1,093	-,094	-,321	,748

* Indicates statistically significant p-Values.

Abbreviations: monocyte-to-lymphocyte ratio; NLR, neutrophil-to-lymphocyte ratio; PIV, pan-immune-inflammation value; PLR, platelet-to-lymphocyte ratio; SII, systemic immune-inflammation index; SIRI, systemic inflammation response index.

To assess the predictors influencing the probability of patients requiring dialysis, we conducted a binary logistic regression. The entered variables were NLR, PLR, MLR, SI, SIRI, and PIV results in ED. MLR and SIRI were statistically significant in forecasting the dialysis requirement with Exp(B) values of 0.306, (p = 0.024) and 1.261, (p = 0.038), respectively **(Table 5).**

**Table 5:** Results of binary logistic regression model performed to predict dialysis requirement.

Variables in the Equation						
	B	S.E.	Wald	df	Sig.	Exp(B)
NLR	,151	,096	2,483	1	,115	1,163
PLR	,000	,002	,003	1	,958	1,000
**MLR**	-1,185	,524	5,117	1	**,024** *	,306
SI	-,001	,000	2,462	1	,117	,999
**SIRI**	,232	,112	4,322	1	**,038** *	1,261
PIV	,000	,000	,011	1	,918	1,000
Constant	-3,471	,369	88,624	1	,000	,031

Abbreviations:, MLR, monocyte-to-lymphocyte ratio; NLR, neutrophil-to-lymphocyte ratio; PIV, pan immune-inflammation value; PLR, platelet-to-lymphocyte ratio; SII, systemic immune-inflammation index; SIRI systemic inflammation response index. B, beta, regression coefficients; S.E., Standard Error, Wald, The Wald tes result; df, degree of freedom; Sig. Significance

* indicates statistically significant p-Values

## Discussion

The present study provides novel insights into how systemic inflammatory indices in patients with earthquake-related crush injuries correlate with the length of hospitalization and dialysis requirement, pioneering the linkage between complete blood count-derived inflammatory markers and outcomes in this unique patient group.

In the aftermath of natural disasters, such as earthquakes, timely and evidence-based decisions in the emergency department (ED) can significantly influence patient outcomes [14]. Our study elucidates the value of utilizing readily available markers in the form of inflammatory indices to predict the duration of hospital stays for earthquake-related crush injury patients. This new insight opens doors to more strategic resource allocation, patient prioritization, and potential patient transfers.

This study’s comprehensive statistical approach aimed to uncover significant predictors for the "duration of hospital stay" in patients with earthquake-related crush injuries. The use of an analysis of variance (ANOVA) revealed salient insights into the relationships between the predictor variables and hospitalization duration.

Firstly, the regression model itself was found to be statistically significant, with a p-value of less than 0.001. This essentially indicates that our selected variables, when considered collectively, offer predictive value for the "duration of hospital stay", better than a model that doesn’t account for these factors.

The constant (or intercept) of the model is estimated to be 41.16, suggesting that the average duration of hospital stay is 41.16 days when all predictor variables are set to zero. However, in practical scenarios, the constant term’s interpretation isn’t always meaningful itself and better understood in the context of other predictors.

NLR (neutrophil-to-lymphocyte ratio) has a positive coefficient but is not statistically significant. This suggests that while NLR increases, the duration of hospital stay might also increase, but this effect is not strong enough to be deemed statistically meaningful. NLR has been identified as a prognostic tool in various clinical scenarios. It’s intriguing to contrast our findings with studies on blunt chest trauma patients, where NLR at ED admission did not predict delayed ARDS in the first five days post- injury [15]. However, it was observed that the NLR was higher in patients with non-focal ARDS. ARDS, a severe lung condition, arises from various etiological factors, including trauma. The nuanced differences in NLR’s predictive value, from our study to the blunt chest trauma context, could be attributed to the distinct nature of injuries. While earthquake-related crush injuries might involve a more widespread systemic inflammatory response due to extensive tissue damage, blunt chest trauma focuses on a specific organ system. Additionally, the nature of ARDS (focal vs. non-focal) might influence the inflammatory response, as suggested by the differential NLR values. This highlights the complexity of using systemic markers in specific injury contexts. In addition, its established association with mortality in critically ill trauma patients aligns with our findings in earthquake-related crush injuries [8]. Neutrophils often surge as an immediate response to acute stress or infection, while lymphocytes, being more chronic responders, may decrease in states of severe systemic stress or immunosuppression. Therefore, a high NLR potentially represents an acute inflammatory state coupled with relative immunosuppression, which can predict worse outcomes in trauma patients. The earthquake-related injuries are, in many ways, unique, given the broad spectrum of potential injuries, from minor contusions to life-threatening crush injuries. The systemic inflammatory response in these patients might be a blend of direct tissue damage, potential ischemia-reperfusion injuries, and subsequent complications. The fact that PLR and MLR were more closely tied to hospitalization duration in our study might suggest that these indices capture this multifaceted response more comprehensively than NLR alone.

PLR (platelet-to-lymphocyte ratio) shows a significant positive effect on the duration of hospital stay. For every unit increase in PLR, the duration of hospital stay increases by approximately 0.058 days. Its statistical significance (p=0.039) emphasizes its potential clinical relevance. This aligns with earlier discussions on PLR’s role in indicating systemic inflammation, which might contribute to slower recovery and hence a longer stay. The prognostic relevance of PLR has been increasingly explored in a variety of clinical settings. While its significance in cancer prognosis has been widely established, our study focuses on its applicability in predicting hospital stays for earthquake victims suffering from crush injuries.

The meta-analysis on the role of PLR in solid tumors showed a consistent relationship between elevated PLR and worse overall survival (OS) across different types of malignancies [9]. Notably, the cut- off values used to define risk groups in that study ranged between 150 and 300 [9]. This complements our findings where a PLR cut-off value of 148.8 demonstrated 65.9% sensitivity and 63.5% specificity. While PLR was associated with overall survival in melanoma patients, its association with progression- free survival was not definitive [16]. This distinction is important. It implies that while PLR might be useful in prognosticating survival outcomes, its role in predicting disease progression remains uncertain.

The melanoma study’s cut-off value for PLR was <120, which differed from the earlier mentioned cut-offs for other conditions [16]. This reinforces the idea that while the systemic inflammatory response is a common thread, its implications and thresholds differ depending on the disease context. While the context is different-oncologic prognosis versus prediction of hospital stay duration-the consistency in cut-off ranges underscores the potential utility of this metric across varied clinical scenarios.

The nuances of how inflammation affects different types of cancer vary, as highlighted by the mentioned meta-analysis. For instance, the strong correlation between PLR and OS in colorectal and ovarian cancers, both of which have ties to systemic inflammation and elevated inflammatory markers [9]. This raises the question of whether systemic inflammatory processes observed in malignancies have parallels in traumatic injuries like those sustained during earthquakes. Indeed, the pathophysiological response to trauma might share inflammatory pathways with malignancies. This could be a potential area for further exploration.

MLR (monocyte-to-lymphocyte ratio) exhibits a significant negative relationship with the duration of hospital stay. The interpretation here is that for every unit increase in MLR, there’s an approximate reduction of 28.639 days in the hospital stay. This means that higher MLR values were associated with shorter hospital stays. This might seem counterintuitive, given that both PLR and MLR represent inflammatory indices, but it underscores the complex interplay of the immune system components in the aftermath of trauma. In the backdrop of earthquake-induced injuries, The ROC analysis presented an MLR cut-off value of 0.28 with commendable sensitivity (84.9%) and specificity (85.5%). The AUC stood impressively at 0.907, signaling its strong predictive prowess.

Wang et all underlined MLR’s potential as a predictor of AKI following heart valve replacement surgery. Patients who developed postoperative AKI had a notably elevated preoperative MLR [17]. The identified cut-off was 0.47, with an AUC of 0.772, underlining the role of systemic inflammation in the onset of postoperative AKI after cardiac interventions.

Jiang et all explored MLR as a prognostic marker for acute kidney injury (AKI) following acute hemorrhagic stroke. A higher MLR was characteristic of patients who developed AKI [18]. The derived MLR cut-off values were 0.5556 (for AKI prediction) and 0.7059 (for in-hospital mortality prediction), implying that systemic inflammation, as denoted by MLR, might play a role in ensuing renal complications and mortality after a stroke.

Earthquake-related crush injuries can be intricate. Beyond the immediate physical trauma, they present an increased risk for AKI [19]. Renal compromise following such traumas, especially AKI, emerges as a concerning sequel. It not only complicates the treatment approach but can escalate to life-threatening scenarios [19, 20]. Recognizing the role of systemic inflammation, as gauged by MLR, offers insights into this renal risk and guides therapeutic strategies, potentially reducing mortality in this patient group. The specific association between MLR and the duration of hospital stay, as showcased in the earthquake injury scenario, provides a tangible metric for patient recovery and prognostication. It becomes particularly significant in light of potential kidney injuries in such patients, emphasizing the need for timely interventions to mitigate morbidity and mortality risks. In our study the binary logistic regression analysis was carried out to ascertain the factors affecting the likelihood of patients requiring dialysis. MLR and SIRI emerged as significant predictors for the requirement of dialysis in patients. (p<0.05)

Number of amputations has a significant positive relationship with the duration of stay. This implies that with each additional amputation, the duration of hospital stay is expected to increase by approximately 15.894 days. Amputations are major surgical interventions, often necessitated by severe injuries. The post-operative recovery, combined with the need for rehabilitation, counseling, and potential complications, can understandably prolong hospitalization.

Other variables like SI, SIRI, PIV, number of injured areas, erythrocyte replacement, platelet replacement, and fresh frozen plasma replacement were found not to be statistically significant predictors for hospital stay. Although SIRI was not found to have significant effect in hospital stay, binary logistic regression analysis revealed that it was an independent factor for the need for dialysis. (Exp(B): 1.261, p= 0.038) The relevance of systemic inflammatory markers goes beyond trauma. PIV has emerged as significant indicators in predicting adverse clinical outcomes following malignancy [21, 22]. Malignancy is known to incite systemic inflammatory responses, with cascading effects on the body’s immune and clotting systems. Elevated PIV might reflect a heightened state of inflammation and immune response, which correlates with worse outcomes. Even they did not have any significant effect in our study, it been shown that SII, SIRI may be effective in determining prognosis in cervical cancer, coronary artery disease and acute coronary syndrome [23, 24]. Understanding their predictive value in such diverse clinical situations underscores the potential universality of these indices as markers of systemic stress and inflammation.

The differential effects of PLR and MLR on hospital stay duration highlight the multifaceted nature of the systemic inflammatory response post-injury. It underscores the importance of not viewing these indices in isolation but rather in the context of the overall clinical picture. The results provide clinicians with potential prognostic tools; for example, a high PLR value upon admission might prompt the healthcare team to anticipate a prolonged hospital stay and plan resources and interventions accordingly. In addition, this highlights the potential of these markers to act as non-invasive, cost- effective, and rapid tools to guide clinical decisions in a disaster-stricken ED setting.

There are some limitations in our study as well. Since it is a single center study, with larger patient groups, the association between earthquake related crush injuries and systemic inflammatory indices can be evaluated more clearly. The study focuses on patients that treated in the Plastic, Reconstructive, and Aesthetic Surgery department, it might not represent the broader population of earthquake victims.

One of the most valuable outcomes of our study is its implications for patient management and hospital logistics during disasters. In locations where medical resources might be stretched thin, and where the priority is to stabilize and potentially transfer the most critical patients to specialized trauma centers, our findings offer a tool to help make those decisions. In situations where advanced imaging and comprehensive evaluations might be time-consuming or unavailable, the inflammatory indices from a CBC can aid in determining the severity of injuries and the potential requirement for extended medical care.

Furthermore, the ability to predict the duration of hospital stay and dialysis requirement can significantly aid in planning and resource allocation. Hospitals can anticipate bed vacancies and optimize the use of their resources based on the projected length of hospital stays for incoming patients. Moreover, our results provide a framework for other hospitals and emergency care facilities to develop protocols for patient triage and potential transfer to specialized centers, ensuring that patients receive the appropriate level of care promptly.

## Conclusion

In the wake of natural disasters, the ability to predict and manage patient outcomes is of paramount importance. Our study stands distinct in its exploration, being the first to directly illustrate the relationship between systemic inflammatory indices and two critical factors: the duration of hospitalization and dialysis requirements in patients with earthquake-related crush injuries. The findings underscore not only the potential application of these indices in assessing earthquake victims but also in managing a myriad of other diseases where inflammatory markers play a significant role. As a pioneering work in this realm, our research introduces a novel, non-invasive, and cost-effective tool. This tool offers immense promise for medical professionals, enabling them to make swift, informed decisions in disaster-stricken environments and beyond. By harnessing the predictive power of inflammatory indices, we can enhance patient care, optimize resources, and ultimately, save lives.

## Supporting information

S1 Data Data of the Study: This dataset from 340 patients (140 with earthquake-related injuries and 200 controls) examines the link between systemic inflammatory indices, hospitalization duration, and dialysis needs post-earthquake. (XLSX)
